# A Study of *Helicobacter Pylori*-associated Gastritis Patterns in Iraq and Their Association with Strain Virulence

**DOI:** 10.4103/1319-3767.48971

**Published:** 2009-04

**Authors:** Nawfal R. Hussein, Sarbar M. Napaki, John C. Atherton

**Affiliations:** 1 Institute of Infection, Immunity and Inflammation, Centre for Biomolecular Sciences, University of Nottingham, Nottingham, and Wolfson Digestive Diseases Centre, Queen's Medical Centre, University of Nottingham, Nottingham, UK; 2 Azadi Teaching Hospital, College of Medicine University of Dohuk, Kurdistan, Iraq

**Keywords:** Antral-predominant gastritis, *H. pylori*, Iraq, low cancer rate

## Abstract

**Background/Aim:**

*Helicobacter pylori* (*H. pylori*) infection causes peptic ulceration and gastric adenocarcinoma. In Iraq, gastric cancer is rare. We investigated whether infected adults had the antral-predominant pattern of *H. pylori*-associated gastritis, which does not predispose to cancer.

**Materials and Methods:**

We evaluated histopathological changes by the Sydney scoring system in gastric biopsies taken from 30 *H. pylori*-infected adults and studied the correlation of these changes with the virulence factors. The Mann-Whitney test was used for the comparison of histopathological data. The presence or absence of each pathological index was evaluated with respect to the possession of virulence factors by the infecting *H. pylori* strain using the χ^2^ test.

**Results:**

Gastric lymphocyte infiltration was more prominent in the antrum (*P* = 0.01). Neutrophil infiltration was mild and gastric mucosal atrophy was rare. No relationship was found between virulence factors and histopathological changes.

**Conclusions:**

The mild pathology and antral-predominant gastritis help explain the low cancer rate in Iraq.

***Helicobacter pylori*** infection causes peptic ulceration and gastric adenocarcinoma. In Iraq, despite the early acquisition of ***H. pylori*** infection,[1] gastric cancer is unusually rare.[2] Despite the geographical proximity of Iraq, Turkey and Iran, the incidence of gastric cancer differs hugely among these countries, being ≤ 5/10^5^, 8.9–14.1/10^5^ and 38–69/10^5^, respectively.[2] Virulence factors of Iraqi ***H. pylori*** strains appear more closely related to Western countries and unlikely to explain the low cancer rate completely.[3] Nothing is known about the distribution of ***H. pylori-*** associated inflammation in Iraqi patients or its severity. Thus, we now aim to assess the degree and distribution of inflammation in the stomachs of Iraqi people and the relationship between ***H. pylori*** virulence factors (***vacA***, ***cagA*** and ***dupA***) and histopathological changes.

## MATERIALS AND METHODS

The study protocol was approved by the ethical committee of the University teaching hospital where biopsies were collected. Samples for histopathology were obtained from 30 adult subjects with dyspepsia and without peptic ulceration. Two biopsies from the antrum and two from the corpus were taken and fixed in 10 mL buffered 10% formalin for histopathological examinations. The histological findings from the sections were scored according to the updated Sydney system of classification and grading of gastritis.[4] These slides were graded for the following features: ***H. pylori*** density, neutrophilic activity, lymphocytic infiltration and glandular atrophy. A visual analogue scale was used to assess the severity of the inflammatory changes and grading was performed as follows: l, mild; 2, moderate; 3, severe. All biopsies had Alcian blue (pH 2.5) to confirm the presence of metaplasia. Because our main aim was to determine the pattern of gastritis, we did not use any special stain to demonstrate ***H. pylori*** and we did not include ***H. pylori*** density in any further analysis.

Polymerase chain reaction-based genotyping of ***H. pylori*** isolates was performed on DNA extracted from bacteria or directly from biopsies.[3]

The Mann–Whitney test was used for the comparison of histopathological data. The presence or absence of each pathological index was evaluated with respect to the possession of virulence factors by the infecting ***H. pylori*** strain using the χ^2^ test.

## RESULTS

Pathological changes were observed in biopsies from 29/30 patients. The biopsies from 1/30 were histologically normal despite ***H. pylori*** being cultured. Among the 29 patients with inflammation in gastric biopsies, lymphocyte infiltration was more prominent in the antrum (Mann–Whitney ***U*** test, ***P*** = 0.01). There was no significant difference in neutrophil infiltration or mucosal atrophy between the antrum and the corpus. However, neutrophil infiltration was mild and was virtually absent (score 0) in 41% of the antral biopsies and 59% of the corpus biopsies. Furthermore, histological evidence of mucosal atrophy was seen in only 1/30 antral biopsies (patient age = 40 years) and 1/30 corpus biopsies (patient age = 64 years). In both the cases, it was mild. Intestinal metaplasia was found in 2/30 (6.8%) and 3/30 (10%) biopsies taken from the antrum and the body, respectively [Table 1].

**Table 1:** *H. pylori*-associated gastritis in biopsies taken from the gastric antrum and corpus

Variables	Antrum				Corpus				*P*-value
			
	**Score 0**	**Score 1**	**Score 2**	**Score 3**	**Score 0**	**Score 1**	**Score 2**	**Score 3**	
Lymphocyte infiltration (*n*)	0	10	14	5	0	20	7	2	0.016*
Neutrophil infiltration (*n*)	12	15	2	0	17	11	1	0	0.19
Intestinal metaplasia (*n*)	27	1	1	0	26	3	0	0	0.68
Atrophy (*n*)	28	1	0	0	28	1	0	0	1

* Significant *P*-value measured by the Mann–Whitney *U* test.

The virulence factors of Iraqi ***H. pylori*** strains and their relationship with clinical outcome were studied previously.[3] In this study, we aimed to explore the relationship between individual virulence determinants and gastric histopathological changes in the gastric antrum and corpus. 66.6% (20/30) and 30% (9/30) of Iraqi strains typed positive for ***cagA*** and ***dupA***, respectively. The distribution of ***vacA*** allelic types among ***H. pylori*** strains is shown in Table 2. For the ***vacA*** s, i and m regions individually, 28/30 (93.3%) isolates were the type s1, 8/30 (26.6%) isolates were the type i1 and 15/30 (23.3%) isolates were the type m1. To test whether the presence of specific genotype correlated with histopathological changes in the antrum and the corpus, our data were restratified according to the histological changes. No significant associations were found between ***vacA***, ***cagA*** and ***dupA*** and histopathological scoring.

**Table 2:** Distribution of the *vacA* allelic types in Iraqi strains

	s1/i1/m1	s1/i1/m2	s1/i2/m1	s1/i2/m2	s2/i2/m2
n (%)	7/30 (23.3)	1/30 (3)	8/30 (26.6)	12/30 (40)	2/30 (6)

## DISCUSSION

***H. pylori*** is a risk factor for gastric cancer and gastric lymphoma.[5] Around the world, despite the high frequency of ***H. pylori*** infection, the incidence of gastric cancer is discordant.[5] The annual incidence rate of gastric cancer is very high in Japan and China, but ***H. pylori*** seropositivity is low.[5] In contrast, in India, seropositivity of ***H. pylori*** is very high but the annual incidence of gastric cancer is low.[5] One possible explanation is the difference in the gastritis pattern as pangastritis and corpus-predominant gastritis are associated with an increased risk of gastric mucosal atrophy and increased risk of cancer.[6,7] In two studies conducted in Japan and Iran, where there is a high gastric cancer rate, it was shown that ***H. pylori*** infection was strongly associated with chronic gastritis and that histological corpus gastritis was found with a high frequency.[8,9] On the other hand, in India and UAE, where the gastric cancer rate is low, the distribution pattern of gastritis was found to be antral-predominant.[10,11] Potentially, the low cancer rate in Iraq could be explained by antral-predominant gastritis being the common pattern and/or by inflammation being mild. In a study conducted in Iran, a neighbouring country to Iraq where the gastric cancer rate is very high,[2] it was found that mononuclear cell infiltration was similar throughout the stomach. On an average, patients had pangastritis.[9] In Iraq, we have shown that there is antral-predominant mononuclear cell infiltration. These findings are in agreement with the results from Kenya, an African country with a very low gastric cancer rate, where there is antral-predominant gastritis with significant discordance in the severity of graded variables between antral and corpus biopsies.[12] Furthermore, in Iran, histological evidence of mucosal atrophy was seen in 39% and 22% of the antral and corpus samples, respectively.[13] In another study conducted in Turkey, it was found that 43% of the ***H. pylori***-infected subjects had atrophic gastritis.[14] In our study, glandular atrophy was found in only one (3%) specimen taken from the antrum and one from the corpus. Thus, despite the early acquisition of ***H. pylori***,[1] the presence of atrophy appears rare in Iraq. We speculate that this antral-predominant gastritis and low glandular atrophy rate in Iraq might contribute to the low cancer rate.

Studies from North America revealed that infection with ***cagA***-positive ***H. pylori*** strains increases the risk of atrophic gastritis and gastric cancer.[15] However, this was not confirmed in several studies in Asian populations.[16] European studies showed a strong significance of the ***vacA*** m1 genotype with respect to epithelial damage, neutrophilic and lymphocytic infiltrates, atrophic gastritis and intestinal metaplasia. In addition, it was shown that severe damage to the gastric epithelium is associated with ***vacA*** s1/m1 mosaicism.[17] In India, it was shown that the s1a/m1 and s1a/m2 ***H. pylori*** vacA genotypes are significantly associated with severe chronic gastritis and gastric epithelial cell apoptosis than s2/m2.[18] In this paper, we aimed to study the correlation between individual virulence markers and histopathological changes in Iraq. In contrast to other studies,[17–19] no correlation between virulence factors and histopathological changes could be observed. In a study conducted in India, risk factors for gastric diseases were assessed in two populations with different incidences of gastric disease. It was shown that diet was the primary factor relating to the differences in the prevalence of duodenal ulcer. In Iraq, virulence factors of ***H. pylori*** were studied but these could not explain the low cancer rate. Probably studying other risk factors such as diet and smoking may help explain the mild pathology.

Our study has limitations, in particular that the sample size is small. However, because of the low rate of atrophy, it is unlikely to be misleading. Why gastric mucosal atrophy is uncommon in Iraq needs further study. However, our results raise the possibility that it may, in part, be due to the antral-predominant infiltration pattern seen in ***H. pylori***-infected Iraqi population.
